# Illness perceptions and self-care behaviors among Chinese hospitalized adults with heart failure

**DOI:** 10.1186/s40359-025-03693-x

**Published:** 2025-12-05

**Authors:** Zehao Huang, Ting Liu, Xi Cao, Sek Ying Chair

**Affiliations:** 1 The Nethersole School of Nursing, Faculty of Medicine, The Chinese University of Hong Kong, Hong Kong SAR, China; 2 School of Nursing, Sun Yat-sen University, Guangzhou, China

**Keywords:** Heart failure, Illness perceptions, Self-care

## Abstract

**Background:**

Illness perceptions, which are representations of illness or health threats formed in response to situational stimuli, are considered one of the most significant predictors of self-care behaviors. However, there is limited evidence regarding the relationships between illness perceptions and self-care behaviors among individuals with heart failure (HF).

**Objective:**

To explore the relationships between illness perceptions and self-care behaviors among Chinese people with HF.

**Methods:**

This is a secondary analysis of baseline data from an intervention program that has received ethical approval and obtained informed consent from all participants. People with HF were recruited from a tertiary hospital in China between February and May 2023. All the participants were assessed for illness perceptions, self-care behaviors, and sociodemographic and clinical characteristics. Hierarchical linear regression analyses were employed to examine the effect of illness perceptions on self-care behaviors.

**Results:**

A total of 164 people with HF were included in the study. Illness perceptions (*β* = -0.215, *p* = 0.002), educational level (*β* = 0.186, *p* = 0.010), employment status (*β* = 0.188, *p* = 0.033), and time since diagnosis (*β* = 0.367, *p* < 0.001) were identified as significant predictors of self-care maintenance. We also found that illness perceptions (*β* = -0.208, *p *= 0.002), educational level (*β* = 0.187, *p* = 0.009), employment status (*β* = 0.223, *p* = 0.002), and time since diagnosis (*β* = 0.375, *p* < 0.001) significantly affected symptom perception. Additionally, illness perceptions (*β* = -0.399, *p* < 0.001), educational level (*β* = 0.213, *p* = 0.003), and time since diagnosis (*β* = 0.142, *p* = 0.039) predicted self-care management.

**Conclusions:**

Illness perceptions are key factors for HF self-care behaviors. Interventions aimed at enhancing illness perceptions may lead to better self-care behaviors.

**Supplementary Information:**

The online version contains supplementary material available at 10.1186/s40359-025-03693-x.

## Introduction

Self-care is broadly defined as the ability of individuals, families, and communities to prevent and manage disease while maintaining and promoting health [1]. Specifically, according to the Situation-specific Theory of Heart Failure (HF) Self-care, HF self-care refers to the process of managing health through self-care maintenance, symptom perception, and self-care management [2]. It is well established that self-care is an effective strategy for the management of HF, as recommended by current healthcare guidelines [3, 4]. Greater engagement in self-care was associated with decreased mortality and hospital readmissions [3, 5, 6], as well as improved patient-centered outcomes such as health-related quality of life [3, 5–9], depression [6, 8, 10, 11], anxiety [8, 10], symptom burden [12], and sleep quality [10].

Despite the well-documented benefits of self-care for HF [3, 4], in several published reports of Chinese patients who participated in similar research, authors found that the performance of self-care was insufficient [13–15]. In a cross-sectional study conducted by Cao et al. [13], the mean scores for self-care maintenance and self-care management were below the recommended cutoff point of 70 for both male and female participants in China. Zou et al. [15] found inadequate self-care among 321 Chinese patients with HF, with mean total scores for self-care maintenance and self-care management of 48.4 and 54.3, respectively. Similar findings were shown in a study by Liu et al. [14], where an average score of 39.71 for self-care maintenance in their sample. These findings underscore the urgent need for interventions to enhance self-care among patients with HF in China.

Identifying the determinants of self-care behaviors is a critical step in designing effective interventions to promote the uptake of self-care. The Common-Sense Model (CSM) of Self-Regulation is a widely used theoretical framework for understanding illness self-care, suggesting that individuals facing health threats form illness perceptions that influence their coping procedures [16]. Illness perceptions, also known as illness representations, are a core concept posited in the CSM of Self-Regulation. They consist of cognitive and emotional representations of illness or health threats as a reaction to situational stimuli, wherein cognitive perceptions (identity, timeline, consequences, personal control, treatment control, illness coherence, and cause) are proposed to regulate objective health threat, while emotional perceptions are regarded as adjusters of negative emotions [17]. Coping procedures are specific behaviors that individuals undertake in response to health threats, such as seeking help from healthcare professionals or monitoring adverse reactions to medication. Based on the CSM, illness perceptions are considered a significant influencing factor in self-care behaviors.

Studies generated inconsistent results regarding the association between illness perceptions and HF self-care behaviors. In a survey conducted in England, consequences, coherence, treatment control, and timeline of illness perceptions were important factors affecting HF self-care behaviors [18]. Similarly, Attard [19] found that illness perceptions were significant predictors of self-care and medication adherence among people with HF in Malta. In contrast, researchers in another study reported no significant relationship between illness perceptions and HF self-care behaviors [20]. In China, a prior study by Mei and colleagues similarly indicated that illness perceptions had no impact on self-care maintenance and self-care management [21]. In summary, the existing evidence regarding the links between illness perceptions and HF self-care behaviors remains equivocal, warranting further investigation to elucidate the nature and extent of these relationships. In addition, several sociodemographic and clinical factors that may affect self-care behaviors among this patient group should be considered when exploring the impact of illness perceptions on self-care behaviors. These factors included age [15, 22], gender [23, 24], marital status [25, 26], educational level [27, 28], employment status [28, 29], place of residence [30], living arrangement [27], New York Heart Association functional Class [29, 31], left ventricular ejection fraction [15, 32], comorbidity [33, 34].

To address the existing research gap, we aimed to examine the relationships between illness perceptions and self-care behaviors among Chinese patients with HF in this study. The research question for this study is: “What are the relationships between illness perceptions and self-care behaviors among people with HF?“.

## Methods

### Design

The present paper is a secondary analysis of baseline data from a HF Self-care Program on patient outcomes. The Strengthening of Reporting of Observational Studies in Epidemiology checklist for cross-sectional studies was followed [35].

### Study setting and sampling

A convenience sample of 164 people with HF was recruited from three cardiology units of a university-affiliated public hospital located in Guangzhou, China between February and May 2023. The principal investigator identified potential participants by reviewing their medical history documented in the hospital information management system. The eligibility of the patients was then assessed based on the inclusion and exclusion criteria. The principal investigator approached the identified patients face-to-face at their bedside to evaluate their willingness to participate in the program, providing each individual with an informed consent form and elaborating its details. Post-hoc achieved-power for liner regression was calculated using G*Power software (“F test, linear multiple regression: fixed model, R2 increase” and “post hoc” power analysis). The hierarchical linear regression for self-care maintenance, symptom perception, and self-care management indicated that illness perceptions explained variances of 0.043 (residual variance: 0.666), 0.042 (residual variance: 0.691), and 0.147 (residual variance: 0.701), respectively. Consequently, the effect sizes for self-care maintenance, symptom perception, and self-care management were 0.065, 0.061, and 0.210, respectively. Considering the respective tested and total number of predictors and an alpha error probability of 0.05, the present sample size achieved sufficient power (all greater than 0.80) [36].

### Inclusion and exclusion criteria

The inclusion criteria were: (1) adults (age ≥ 18 years); (2) clinically diagnosed with heart failure; (3) classified as New York Heart Association functional class I-IV; (4) able to complete the questionnaire; (5) able to communicate in Mandarin or Cantonese; and (6) agree to participate in the study. Patients were excluded if they had cognitive impairment, psychiatric disease, severe physical illness, or had recently been involved in other experimental studies.

### Outcome measures and data collection

Before data collection, all eligible participants were informed of the purpose of the study and asked to sign the informed consent form. After obtaining informed consent, the principal investigator collected data in the patient’s room. During the process of filling out the questionnaire, uniform explanations were provided for each item to assist participants in accurately filling it out. After patients completed the questionnaire, the principal investigator reviewed the results to ensure that no data was missing. We have received approval to use the 29-item Self-Care of Heart Failure Index version 7.2, while no approval was required for the use of the 9-item Brief Illness Perception Questionnaire.

#### Self-care behaviors

HF self-care behaviors were measured using the 29-item Self-Care of Heart Failure Index version 7.2 [37]. The scale was divided into three subscales: self-care maintenance (10 items), symptom perception (11 items), and self-care management (8 items) [37]. Each subscale is scored separately and scores on each subscale are summed and standardized to a 100-point scale, with higher scores indicating better self-care [37]. A score ≥ 70 represents adequate self-care and an 8-point difference in the standardized score indicates a minimal clinically important difference [37]. The Chinese version scale is valid and reliable [38]. Cronbach’s coefficients of self-care maintenance, symptom perception, and self-care management were 0.79, 0.89, and 0.77, respectively; and their test-retest reliability was 0.76, 0.78. and 0.75, respectively [38].

#### Illness perceptions

The 9-item Brief Illness Perception Questionnaire was used to evaluate the cognitive and emotional representations of illness in people with HF [39]. This scale consists of 7 items to assess cognitive representations of illness, 6 of which are used to measure identity (item 5), consequences (item 1), personal control (item 3), treatment control (item 4), timeline (item 2), coherence (item 7), and an open-ended question exploring the causes (item 9). Moreover, 2 items are used to assess emotional representations: concern (item 6) and emotions (item 8) [39]. When calculating the total scores, the scores for items 3, 4, and 7 need to be reversed [39]. Items are rated using a Likert scale ranging from 0 ~ 10 [39]. The total score ranges from 0 to 80, with lower total scores indicating better illness perceptions [39]. This instrument had good test-retest reliability, concurrent validity, predictive validity, and discriminant validity. Mei et al. [40] translated and validated the Chinese version scale (Cronbach’s ɑ coefficient was 0.77 and split-half reliability was 0.81).

#### Sociodemographic and clinical characteristics

Sociodemographic data included age, gender, marital status, educational level, employment status, place of residence, and living arrangement. Clinical information gathered comprised the time since diagnosis, New York Heart Association functional Class, left ventricular ejection fraction, comorbidity measured by age-adjusted Charlson Comorbidity Index [41], and N-terminal B-type natriuretic peptide. All sociodemographic and medical data were extracted from the participants’ electronic medical records during the enrollment period using a self-designed questionnaire.

### Data analysis

The statistical assumption of the normality of continuous variables was examined by quantile-quantile plots and skewness and kurtosis statistics. Normal distribution of the data was assumed if the data points were close to the straight diagonal line in the normal quantile-quantile plots. Additionally, values for skewness and kurtosis between − 2 and + 2 are considered acceptable indicators of normality [42, 43]. Normally distributed continuous variables were presented in means and standard deviations (SDs). Variables that did not achieve normality were described as medians and quartiles (P25, P75). All categorical variables were presented as frequencies and percentages. Independent t-tests, one-way analysis of variance, Pearson’s correlation analysis (*r*), or Spearman correlation analysis (*r*_*s*_) were used to identify potential factors correlated to self-care behaviors. Hierarchical linear regression analyses were executed to test the relationships between illness perceptions and self-care behaviors. Patient characteristics that exhibited statistical differences in self-care behaviors during the univariate analysis were included in Step 1 as confounding factors. In Step 2, illness perceptions were added as the independent variable. We assessed multicollinearity using tolerance and variance inflation factors (VIF), with acceptable criteria set at a tolerance greater than 0.1 and a VIF less than 10 [44]. The statistical analyses were conducted using IBM SPSS version 27.0. The threshold for statistical significance was set at *p* < 0.05 (two-tailed).

## Results

### Participant characteristics, illness perceptions, and self-care behaviors

The skewness and kurtosis values for age, left ventricular ejection fraction, Age-adjusted Charlson Comorbidity Index, illness perceptions, and self-care behaviors ranged from − 2 to 2, implying that these variables were normally distributed. Additionally, the normal distribution of these variables was confirmed by the data points being close to the straight diagonal line in the quantile-quantile plots.

A total of 164 individuals with HF were recruited for the present study. The mean age of the sample was 63.36 years (SD = 12.20; range, 30 to 84 years). Most participants were male (70.1%) and unemployed (79.9%) and had completed primary or secondary education (84.7%). Nearly all the participants were married (97.0%) and lived with others (95.7%), with over half (59.1%) residing in the cities. Among participants, the average left ventricular ejection fraction was 43.24% (SD = 16.33), 60.4% were classified as New York Heart Association functional class Ⅲ to Ⅳ, and the mean age-adjusted Charlson Comorbidity Index was 3.78 (SD = 1.63). The reported median duration after diagnosis of HF was one month (1.00, 11.50) and the median N-terminal B-type Natriuretic Peptide was 2414.00 (1043.25, 5961.75). Characteristics of the participants are summarized in Table 1.

**Table 1:** Participant characteristics, illness perceptions, and self-care behaviors (*n* = 164)

Variables	Mean ± SD	*N* (%)	Median (P25, P75)
Age (years, range 30–84)	63.36 **±** 12.20		
Gender			
Male		115 (70.1)	
Female		49 (29.9)	
Marital status			
Married		159 (97.0)	
Others		5 (3.0)	
Educational level			
Primary or less		52 (31.7)	
Secondary		87 (53.1)	
Tertiary or above		25 (15.2)	
Employment status			
Employed		33 (20.1)	
Unemployed		131 (79.9)	
Place of residence			
Urban		97 (59.1)	
Rural		67 (40.9)	
Living arrangement			
Living alone		7 (4.3)	
Living with others		157 (95.7)	
Time since diagnosis (month)			1.00 (1.00, 11.50)
Left ventricular ejection fraction (%)	43.24 ± 16.33		
New York Heart Association functional classification			
Class Ⅰ to Ⅱ		65 (39.6)	
Class Ⅲ to Ⅳ		99 (60.4)	
Age-adjusted Charlson Comorbidity Index	3.78 ± 1.63		
N-terminal B-type Natriuretic Peptide (pg/ml)			2414.00 (1043.25, 5961.75)
Illness perceptions	45.98 ± 8.29		
Self-care maintenance	22.79 ± 9.12		
Symptom perception	21.59 ± 11.76		
Self-care management	42.17 ± 10.38		

As shown in Table 1, participants exhibited a moderate level of illness perceptions, with a mean score of 45.98 (SD = 8.29). Regarding self-care behaviors, self-care management demonstrated the highest mean score of 42.17 (SD = 10.38), followed by self-care maintenance with a mean score of 22.79 (SD = 9.12), while symptom perception had the lowest average score of 21.59 (SD = 11.76).

### Relationships among patient characteristics, illness perceptions, and self-care behaviors

The results indicated that educational level (*F* = 7.641, *p* < 0.001), employment status (*t* = −2.542, *p* = 0.012), place of residence (*t* = 2.466, *p* = 0.015), age (*r* = 0.198, *p* = 0.011), and time since diagnosis (*r*_*s*_ = 0.428, *p* < 0.001) were significantly associated with self-care maintenance. Educational level (*F* = 6.150, *p* = 0.003), employment status (*t* = −2.670, *p* = 0.008), and time since diagnosis (*r*_*s*_ = 0.561, *p* < 0.001) were found to correlate with symptom perception. In addition, educational level (*F* = 12.045, *p* < 0.001), place of residence (*t* = 2.463, *p* = 0.015), time since diagnosis (*r*_*s*_ = 0.183, *p* = 0.019), and N-terminal B-type Natriuretic Peptide (*r*_*s*_ = −0.181, *p* = 0.021) were related to self-care management. The mean scores of illness perceptions were negatively associated with the mean scores of self-care maintenance (*r* = −0.315, *p* < 0.001), symptom perception (*r* = −0.295, *p* < 0.001), and self-care management (*r* = −0.467, *p* < 0.001). (see Table 2).

**Table 2:** Relationships among patient characteristics, illness perceptions, and self-care behaviors

Variables	Self-care maintenance		Symptom perception		Self-care management	
	Mean ± SD	t/F/*r*/rs	Mean ± SD	t/F/*r*/rs	Mean ± SD	t/F/*r*/rs
Age (years)		0.198*		0.152		0.049
Gender		−0.669		0.117		−0.158
Male	22.48 ± 9.64		21.66 ± 12.07		42.08 ± 10.39	
Female	23.52 ± 7.80		21.43 ± 11.10		42.36 ± 10.45	
Marital status		−0.052		−0.196		0.088
Married	22.78 ± 9.16		21.56 ± 11.85		41.95 ± 10.14	
Others	23.00 ± 8.55		22.61 ± 9.30		49.09 ± 16.23	
Educational level		7.641**		6.150**		12.045**
Primary or less	21.73 ± 8.95		20.32 ± 11.11		39.57 ± 9.40	
Secondary	21.61 ± 7.89		20.24 ± 10.80		41.24 ± 9.43	
Tertiary or above	29.10 ± 11.08		28.96 ± 13.86		50.79 ± 11.38	
Employment status		−2.542*		−2.670**		−0.351
Employed	19.24 ± 8.58		16.80 ± 10.58		41.60 ± 10.77	
Unemployed	23.68 ± 9.06		22.80 ± 11.77		42.31 ± 10.31	
Place of residence		2.466*		1.677		2.463*
Urban	24.23 ± 9.46		22.81 ± 12.70		43.80 ± 10.69	
Rural	20.71 ± 8.23		19.82 ± 10.07		39.80 ± 9.49	
Living arrangement		−0.826		−1.472		0.292
Living alone	20.00 ± 8.54		15.22 ± 10.35		43.29 ± 15.02	
Living with others	22.91 ± 9.15		21.88 ± 11.77		42.12 ± 10.19	
Time since diagnosis (month)		0.428**		0.561**		0.183*
Left ventricular ejection fraction (%)		0.077		−0.110		0.096
New York Heart Association functional classification		−0.161		−0.756		0.631
Class Ⅰ~Ⅱ	22.65 ± 7.95		20.74 ± 11.74		42.80 ± 9.26	
Class Ⅲ~Ⅳ	22.88 ± 9.85		22.16 ± 11.80		41.75 ± 11.08	
Age-adjusted Charlson Comorbidity Index		0.150		0.051		−0.102
N-terminal B-type Natriuretic Peptide (pg/ml)		0.018		0.076		−0.181*
Illness perceptions		−0.315**		−0.295**		−0.467**

*t* Independent t-tests, *F* one-way analysis of variance, *r* Pearson’s correlation analysis, *rs* Spearman correlation analysis

*p < 0.05

**p < 0.01

### Hierarchical linear regressions of self-care behaviors

No multicollinearity was detected in the present study, as tolerance values were above 0.1 and VIF values were below 10. After controlling significant characteristics of participants, the results indicated that higher mean scores of illness perceptions were significantly associated with lower mean scores in self-care maintenance (*β* = −0.215, *p* = 0.002), symptom perception (*β* = −0.208, *p* = 0.002), and self-care management (*β* = −0.399, *p* < 0.001). Educational level, employment status, time since diagnosis, and illness perceptions accounted for 33.4% (R^2^ = 0.334) of the variance in self-care maintenance, with illness perceptions contributing 4.4% (ΔR^2^ = 0.044) of the explained variance. For symptom perception, educational level, employment status, time since diagnosis, and illness perceptions explained 30.9% (R^2^ = 0.309) of the variance, with illness perceptions accounting for 4.2% (ΔR^2^ = 0.042) of this total. Additionally, educational level, time since diagnosis, and illness perceptions accounted for 29.9% (R^2^ = 0.299) of the variance in self-care management, with illness perceptions contributing to 14.6% (ΔR^2^ = 0.146) of the explained variance. Table 3 presents the results of the regression analysis of self-care behaviors.

**Table 3:** Hierarchical linear regressions of self-care behaviors

	Variable	Model 1		Collinearity statistics		Model 2		Collinearity statistics	
		β	*p*	Tolerance	VIF	β	*p*	Tolerance	VIF
Self-care maintenance	(Constant)		0.145				0.002		
	Educational level	0.214	0.004	0.847	1.180	0.186	0.010	0.834	1.199
	Employment status	0.202	0.026	0.556	1.799	0.188	0.033	0.554	1.804
	Place of residence	−0.107	0.138	0.872	1.147	−0.080	0.255	0.859	1.164
	Age	0.058	0.520	0.563	1.777	0.054	0.533	0.563	1.778
	Time since diagnosis	0.388	< 0.001	0.963	1.038	0.367	< 0.001	0.953	1.049
	Illness perceptions					−0.215	0.002	0.943	1.061
	*R*^*2*^*/adjusted R* ^*2*^ *(ΔR*^*2*^*)*	0.290/0.268 (0.290)				0.334/0.308 (0.044)			
	*F*	12.916**				13.096**			
Symptom perception	(Constant)		0.925				0.032		
	Educational level	0.221	0.002	0.902	1.108	0.187	0.009	0.881	1.135
	Employment status	0.238	0.001	0.908	1.102	0.223	0.002	0.903	1.107
	Time since diagnosis	0.398	< 0.001	0.976	1.025	0.375	< 0.001	0.964	1.038
	Illness perceptions					−0.208	0.002	0.958	1.044
	*R*^*2*^*/adjusted R*^*2*^ *(ΔR*^*2*^*)*	0.267/0.253 (0.267)				0.309/0.291 (0.042)			
	*F*	19.440**				17.751**			
Self-care management	(Constant)		< 0.001				< 0.001		
	Educational level	0.245	0.002	0.898	1.113	0.213	0.003	0.893	1.120
	Place of residence	−0.101	0.186	0.925	1.081	−0.052	0.459	0.911	1.097
	Time since diagnosis	0.195	0.010	0.963	1.038	0.142	0.039	0.946	1.057
	N-terminal B-type Natriuretic Peptide	−0.111	0.139	0.956	1.046	−0.046	0.506	0.930	1.075
	Illness perceptions					−0.399	< 0.001	0.923	1.084
	*R*^*2*^*/adjusted R*^*2*^ *(ΔR*^*2*^*)*	0.153/0.131 (0.153)				0.299/0.277 (0.146)			
	*F*	7.169**				13.510**			

*VIF* Variance Inflation Factor

**p < 0.01

## Discussion

This study investigated the relationship between illness perceptions and self-care behaviors in individuals with HF. We found that better illness perceptions were significant predictors of improved self-care behaviors, offering valuable insights for enhancing the uptake of self-care practices among people with HF.

The mean score of illness perceptions in the current study was similar to the results of previous studies in Chinese patients with HF [45, 46]. In contrast to the study by Mei et al. [21], our study indicated that illness perceptions were significant predictors of self-care behaviors among Chinese patients with HF. This discrepancy may be due to the inclusion of self-care confidence and self-care management as independent variables in the prior study [21]. Participants who had more threatening views of HF demonstrated diminished self-care maintenance, symptom perception, and self-care management. This finding is supported by the CSM of Self-Regulation, which posits that an individual’s perceptions of health threats affect their coping behaviors [17]. It is suggested that 0.005, 0.01, 0.02, and 0.09 represent small, medium, large, and very large cut-offs for R² (regression), respectively [47–52]. In this study, illness perceptions explained 4.4% variance of self-care maintenance and 4.2% variance of symptom perception, both of which exceeded the large effect size benchmark (2%). Moreover, illness perceptions accounted for 29.9% variance of self-care management, surpassing the large effect size benchmark of 9%. The development of illness perceptions are shaped by both internal and external factors, such as experienced symptoms and signs, messages presented in pamphlets, and information provided by others [17]. Patients’ beliefs about HF have a great impact on their motivation to engage in proactive health behaviors, such as adhering to medication regimens, restricting sodium intake, exercising, monitoring symptoms and signs, and managing worsening conditions [19, 53]. Therefore, healthcare professionals should prioritize assessing patients’ illness perceptions and provide effective strategies to improve their beliefs about the condition, thereby enhancing their engagement in HF self-care practices. For instance, medical workers can utilize diverse educational instruments, such as videos and illustrations, to connect different causes and impacts related to HF, which can improve the patients’ understanding of the causes and prognosis and strengthen their illness identity [54–56]. Furthermore, to improve patients’ sense of personal and treatment control over HF, clinical staff can offer tailored self-care information, help them develop personal goals and address self-care barriers, and teach them use problem-solving skills to facilitate decision-making [54–56].

The present study also contributes to the literature by identifying several sociodemographic and clinical characteristics including educational level, employment status, and time since diagnosis that can predict self-care behaviors. The findings indicated that individuals with higher educational levels, those who were unemployed, and those with longer HF durations were more likely to engage in self-care maintenance and symptom perception practices. Additionally, individuals with higher education and unemployment status exhibited better self-care management. Several studies have yielded consistent evidence that higher education levels were linked to improved HF self-care behaviors [27, 28, 33]. Ryou et al. [22] found that being employed was predictive of poor provider-directed adherence. Moreover, some investigations have demonstrated a positive relationship between HF duration and self-care behaviors [15, 27]. Patients with higher education levels typically exhibit improved health literacy, enabling them to better understand medical information and the importance of taking care of themselves, as well as greater motivation to adhere to self-care routines [57]. Diminished self-efficacy may account for the challenges in practicing self-care at work. Individuals who are less occupied with work or other tasks often have more free time to focus on self-care activities, such as exercise, meal preparation, monitoring symptoms, and attending medical appointments [58]. Those with a longer duration of HF often gain more experience and knowledge about maintaining stability and monitoring symptoms, which prompts more proactive engagement in self-care [28]. Given these insights, healthcare providers should offer additional support to individuals with lower educational levels, those who are employed, and those with a short duration of HF.

In our study, we observed suboptimal self-care behaviors, with scores across all three domains—self-care maintenance, symptom perception, and self-care management—measured by the Self-Care of Heart Failure Index version 7.2 falling below the 70-point threshold. The average score for self-care maintenance was lower than the results of previous studies in Chinese patients with HF [13–15]. This discrepancy might be attributed to the fact that many participants were newly diagnosed with HF in our study, resulting in insufficient information about sustaining the stability of physiological health. Additionally, variations in measurement tools could influence the results, as the behaviors assessed in Self-Care of Heart Failure Index version 6.2 differ from those in Self-Care of Heart Failure Index version 7.2. With respect to self-care management, a study reported poorer HF self-care management compared to ours [13], probably because their participants had advanced cardiac function (more participants classified as NYHA Ⅲ and Ⅳ), which might have led to a diminished perceptions of the benefits of self-care management. Conversely, the other study indicated better self-care management in Chinese patients with HF [15]. The reason for poorer self-care management in this study may be due to a shorter duration of HF in our sample, leading to less experience and knowledge about managing the onset of HF-related symptoms. The variations in measurement tools might also contribute to the different results. Symptom perception was scored lowest in the current sample, as most participants had new-onset HF and thus had less knowledge and skills about symptom detection and monitoring.

This study has several limitations. First, the use of cross-sectional data prevents us from establishing causal relationships between illness perceptions and HF self-care behaviors. Thus, future research should consider longitudinal and experimental designs. Second, the representativeness of our sample may have been compromised, as participants were enrolled from a single general hospital in Mainland China, primarily consisting of newly diagnosed patients with HF patients and exhibiting an unbalanced gender distribution. Future studies are encouraged to replicate this research with more diverse populations and a balanced gender representation to enhance the generalizability of the findings. Third, the study outcomes were measured using self-reported questionnaires, which may be subject to social desirability bias. Last, contributions of caregivers to patient self-care may be a confounding factor that was not evaluated in the current study.

## Conclusion

The present study found that illness perceptions are crucial determinants of HF self-care behaviors. Our findings suggest that interventions focused on improving illness perceptions could enhance self-care activities among people with HF.

## Supplementary Information

Supplementary Material 1.

## Data Availability

Data cannot be shared openly but are available on request from the corresponding author (sychair@cuhk.edu.hk).

## Abbreviations

CSM: Common-Sense Model
HF: Heart failure
NYHA: New York Heart Association
SD: Standard deviation
VIF: Variance inflation factors
